# An Overview of Myeloid Blast-Phase Chronic Myeloid Leukemia

**DOI:** 10.3390/cancers16213615

**Published:** 2024-10-26

**Authors:** Gulsum E. Pamuk, Lori A. Ehrlich

**Affiliations:** Office of Oncologic Diseases, Center for Drug Evaluation and Research—CDER, U.S. Food and Drug Administration, 10903 New Hampshire Avenue, Silver Spring, MD 20993, USA; lori.ehrlich@fda.hhs.gov

**Keywords:** myeloid blast-phase (crisis) chronic myeloid leukemia, biology of myeloid blast-phase (crisis) chronic myeloid leukemia, treatment of myeloid blast-phase (crisis) chronic myeloid leukemia, resistance to tyrosine kinase inhibitors

## Abstract

Myeloid blast-phase chronic myeloid leukemia (MBP-CML) is a rare disease with a dismal prognosis. It is twice as common as lymphoid blast-phase CML, and its prognosis is poorer. Despite the success with tyrosine kinase inhibitors in the treatment of chronic-phase CML, the same does not hold true for MBP-CML. In addition to the Philadelphia chromosome, other chromosomal and molecular changes characterize rapid progression. Although some progress in elucidating the biology of MBP-CML has been made, there is need to discover more in order to develop more satisfactory treatment options. Currently, most common treatment options include tyrosine kinase inhibitors (TKIs) as monotherapy or in combination with acute myeloid leukemia-based intensive chemotherapy regimens. Some patients may develop resistance to TKIs via BCR-ABL1-dependent or BCR-ABL1-independent mechanisms. In this paper, we provide an overview of the biology of MBP-CML, the current treatment approaches, and mechanisms of resistance to TKIs. In order to improve treatment responses in these patients, more emphasis should be placed on understanding the biology of myeloid blastic transformation in CML and mechanisms of resistance to TKIs. Although patient numbers are small, randomized clinical trials should be considered.

## Simple Summary

Treatment outcomes in chronic myeloid leukemia (CML) have improved in recent years because of the use of tyrosine kinase inhibitors. However, treatment responses to myeloid blast-phase CML (MBP-CML) are still unsatisfactory. In this paper, we summarize the biological mechanisms of progression to MBP and the most frequently used treatment regimens for MBP-CML. We also review why some patients develop resistance to tyrosine kinase inhibitors.

## 1. Introduction

Chronic myeloid leukemia (CML) is a rare myeloproliferative neoplasm with an incidence of 1 to 2 per 100,000 new patients per year [1]. The disease is characterized by the Philadelphia chromosome (Ph), a reciprocal translocation between chromosomes 9 and 22, and results in the oncogenic fusion protein BCR-ABL1 that acts as a constitutively active tyrosine kinase [2].

Traditionally, CML has three phases. Most patients (85–90%) present with chronic-phase (CP) disease, characterized by an elevated white blood cell count that consists of myeloid precursors and mature cells, and used to last 3–5 years before the use of tyrosine kinase inhibitors (TKIs) [3,4]. About 4–5% of patients present with accelerated-phase (AP) CML, in which there are additional genetic mutations, increase in blasts, and cytopenias. About 1–2% of patients present with blast-phase (BP) CML (also known as “blast crisis” CML) [5]. Approximately 20–25% of patients with BP-CML are diagnosed at initial diagnosis, which is called de novo BP [6]. In the ELN Blast Phase Registry, which includes 240 evaluable patients, BP evolved from previous CML in 151 patients (63%) and occurred as de novo BP in 89 patients (37%) [7]. The routine use of TKIs for the treatment of CP-CML has improved disease outcomes significantly, and most patients can now have near-normal life spans [8]. Furthermore, the risk of transformation to AP- or BP-CML has decreased from 5–20% to 1–5% per year [9]. Nevertheless, BP-CML is a serious disease, and there is unmet medical need for better treatment options.

BP-CML can have different phenotypes, including myeloid, lymphoid, mixed, and megakaryoblastic [10,11]. Most patients with BP-CML (70–80%) present with myeloid blast-phase CML (MBP-CML), and 20–30% are diagnosed with lymphoid blast-phase CML (LBP-CML) [12]. LBP-CML generally has better outcomes than MBP-CML [11].

In this review, we discuss the biology of MBP-CML, the current treatment approaches, and the possible mechanisms of resistance to the current treatments.

## 2. Definition of Myeloid Blast-Phase CML

There are multiple classification systems that are used to define AP- and BP-CML. These are the International Blood and Marrow Transplant Registry (IBMTR) [13], M. D. Anderson Cancer Center (MDACC) [14], European LeukemiaNet (ELN) [15], and the World Health Organization (WHO) criteria [16]. One of the main differences between these classification systems is the blast percentages used to define AP- and BP-CML. According to the IBMTR, MDACC, and ELN, the threshold to define BP is ≥30% blasts in the peripheral blood or bone marrow (BM). Alternatively, the 2022 WHO classification describes BP as ≥20% blasts in either of these locations, omitting the category of AP-CML. Thus, while the WHO classifies patients with 20–30% blasts as BP, these continue to be considered AP by the other systems. All four systems include extramedullary blast proliferation as indicative of BP. For BP-CML, IBMTR criteria are more frequently used than others and WHO criteria are used less [17]. When evaluating results from different clinical trials, the differences between the criteria should be considered, and trial results should be interpreted accordingly.

It is important to determine the cell lineage of BP as this will determine the treatment approach. Clinical and laboratory features should be considered together with the cytogenetic profile [6]. 

TP53 mutations and isochromosome i(17q) are more frequent with MBP-CML, and LBP-CML is more commonly associated with hypodiploidy, monosomy 7, and CDKN2A mutations [18]. Flow cytometry and immunohistochemistry are also used to determine the cell lineage.

## 3. Prognostic Risk Factors in Myeloid Blast-Phase CML

There are certain clinical, cytogenetic, and molecular risk factors that may signify a poor prognosis in BP-CML. Some of these clinical risk factors are age (≥58 years), high serum LDH (≥1227 IU/L), bone marrow blasts (≥5%), anemia (<13 g/dL), thrombocytopenia (<102,000/mm^3^), myeloid immunophenotype, prior TKI usage, and progression from CP- or AP-CML. In this study, the 5-year survival rate in patients with MBP-CML was 15% compared to 30% in patients with LBP-CML. The authors used a Classification and Regression Tree analysis to identify the cutoff values for continuous variables that correlated with survival outcomes [19].

The German CML IV Study was a prospective randomized study of several imatinib treatment regimens and included data from 1151 Ph+ CML patients. Long-term observational data are available from this study, and the presence of additional cytogenetic abnormalities (ACAs) (second Ph chromosome, trisomy 8, i(17q), or trisomy 19) was associated with the progression of CML to AP or BP. Their presence also had a negative effect on overall survival (OS) [20]. One study defined 3q26.2 rearrangement as a change that correlated with a high frequency of ABL1 mutations and proposed that TKI resistance was important in progression to BP. In MBP, trisomy 8, 3q26.2 rearrangement, and i(17q) were significantly more frequent [21].

## 4. The Biology of Blast-Phase CML

CP-CML is characterized by the Ph chromosome, and TKIs, which target the BCR-ABL fusion protein, led to significantly improved outcomes for these patients [22]. However, BC is different from CP-CML in many aspects because it is characterized by additional chromosomal and molecular secondary changes [23]. These changes lead to increased proliferation and survival, and a block in differentiation and apoptosis [23,24]. The accumulation of these changes may be responsible for the unsatisfactory treatment results in BC [24]. Although there clearly is not just one driver that fuels progression to BC, it might be thought of as a consequence of the interplay of multiple mechanisms that are centered around the constitutive overactivity of BCR-ABL1 [11,22].

Prior to the phenotypic transformation of the malignant clone, it was shown that there was an increase in BCR-ABL1 mRNA expression in leukemia cells from patients with CP-CML who progressed to AP or BP [25]. In addition, cells that express high levels of BCR-ABL1, similar to the situation in BC, were found to be much less sensitive to imatinib. The emergence of a mutant subclone resistant to imatinib occurred in a much shorter time in these cells than in cells with low BCR-ABL1 expression, similar to the situation in CP [26]. Increasing BCR-ABL1 levels with disease progression stimulate the production of reactive oxygen species (ROS) leading to BCR-ABL1 DNA damage and inefficient DNA damage repair mechanisms at the level of leukemic stem cells (LSCs) and/or leukemic progenitor cells [27,28]. This, in turn, causes resistance to treatment with TKIs and genomic instability [29].

We will discuss the various mechanisms that are implied in the progression to BC in general and include information specific for MBP-CML when available. Select biologic events are summarized in Table 1.

### 4.1. Genetic Events

Patients who have BCR-ABL1 kinase domain mutations have a higher likelihood of progression to BC. Liu et al. analyzed the incidence of mutations in the BCR-ABL1 kinase region in 175 patients with newly diagnosed or treated CML. Twenty-eight different mutations were detected in fifty-four of the patients (30.9%). T315I mutation was detected in 14 patients (8%). There was more than one mutation in eight patients (4.6%) [30]. Soverini et al. detected P-loop mutations (G250E, Y253F, Y253H, E255K, E255V) in 9 of 40 patients with late CP-CML. These patients had significantly worse outcomes in terms of time to progression and survival. Some P-loop mutations like Y253F and E255K have shown oncogenic activity and enhanced tyrosine phosphorylation [31].

It was reported that the genetic or functional inactivation of p53 seemed the most common abnormality in BC-CML and that the *TP53* gene was mutated in 25–30% of patients with MBP-CML [23]. The effect of p53 restoration in CML primitive/stem cells was studied in a mouse model of CML. The transient restoration of p53 in these mice delayed disease progression and led to a much longer lifespan. Sca1^+^ cells, which express the oncogene, were killed after the restoration of p53 induced the expression of pro-apoptotic target genes [32].

It is known that there may be high expression of the EVI1 transcription factor in MBC; however, a clear mechanism has not been proposed. Manachai et al. reported that enhanced EVI1 expression in BC-CML was dependent on events causing the activation of the LEF1/β-catenin complex and loss of p53 function. They observed that a low p53 expression was mainly present in MBC-CML cell lines. The β-catenin/EVI1 axis shows a poor prognosis in BC-CML and indicates chemotherapy resistance [33].

Ikaros is a zinc finger protein and transcription factor that is encoded by the *IKZF1* gene. Ikaros acts as a regulator of hematopoiesis. *IKZF1* deletions were known to be associated with poor survival in acute lymphoblastic leukemia (ALL); but it has also been identified in both MBC and LBC. In one study, they determined mRNA expression of IKZF1 isoforms in Ph+ leukemia. IK6or IK10was detected when there was progression from CP to BC when there was a simultaneous increase in the BCR-ABL1 transcript level. *IKZF1* non-DNA-binding short-form *Ik6*/*Ik10* transcripts were more frequent in LBC than in MBC [34].

c-MYC induces growth-factor-independent proliferation in CML and is one of the key mediators of leukemic progression [35]. An elevated c-MYC expression increases the risk of progression to BC, and TKI-resistant CML cells may be targeted by inhibiting c-MYC [23,24]. BET inhibitors downregulate the expression of c-MYC, induce apoptosis and differentiation, and suppress the proliferation of cancer cells. They were able to eliminate LSCs in AML models; and it is thought that they can eliminate CML LSCs when combined with TKIs [36].

RUNX1 (also referred to as CBFA2) is an important component of the core binding factor complex. *RUNX1* mutations were detected in several hematologic malignancies, including CML. Using whole-exome and RNA sequencing, the mutational profiles of *RUNX1*-mutated and wild-type BP-CML patients were investigated. The authors showed that CD19-CAR T cells had a potent ex vivo cytotoxic targeting against *RUNX1*-mutated blasts in both MBC and LBC [37].

GATA family proteins serve important roles in the development of some cells. GATA 1, 2, and 3 are required for normal hematopoiesis. The acquired mutations of *GATA2*, and especially its gain-of-function mutation (p.L359V), can be encountered in BP-CML [38]. However, it could not be detected in any patients with CP-CML [39].

Neviani et al. found that the phosphatase activity of the tumor suppressor PP2A was significantly impaired in CD34+ MBC compared to a modest impairment in CD34+ CP-CML. SET is a physiological inhibitor of PP2A, and when induced by BCR-ABL, in a dose- and kinase-dependent manner, it inactivates PP2A. If PP2A activity is re-established in CD34+ myeloid BC cells, there will be a marked inhibition of proliferation and induction of apoptosis, and this may be used as a treatment strategy in BC [40].

It was hypothesized that UBE2A has a role in the regulation of transcription and chromatin reorganization. Magistroni et al. reported that the *UBE2A* mutations analyzed in their study decreased the activity of the enzyme. CSF3R (also known as granulocyte colony-stimulating factor receptor, GCSFR) has a role in promoting neutrophilic differentiation in addition to possibly supporting the development of various hematopoietic progenitors [41,42]. The authors’ data suggest that UBE2A may be mediating CSF3R regulation at the transcriptional level, which may lead to the suppression of myeloid differentiation in BC. This study showed that *UBE2A* mutations were acquired during the progression of CML, and the frequency reached 16.7% in advanced cases [43].

In one study, the gene expression profiles of malignant cells from 11 patients with newly diagnosed CP- and 9 BP-CML were studied. It was observed that SOCS2 had the largest increase in the transcript lemyvel. It is active in the Ras and PI-3 kinase signal transduction cascades and is overexpressed in the advanced stages of CML in a *bcr-abl-*dependent manner [44]. There were also increased transcript and protein levels for CD52 in BP-CML. CD52 is not expressed on the surfaces of most myeloid cells, and its upregulation in BC suggests that CD52 might be a marker of leukemic progression [44,45]. On the other hand, *MPO* was the gene with most decreased transcript levels in BC. This may be expected given that there is a perturbation of myelopoiesis in CML and MPO deficiency is an intrinsic property of blasts in all phases of CML [44,46].

Zheng et al. demonstrated that the levels of the MHC class II complex-related genes *HLA-DRB1, HLA-DRA, HLA-DPA1,* and *CD74* were elevated in BC. CD34 has essential functions in early hematopoiesis, and cells from patients with BC had elevated levels of *CD34* transcripts. They also reported the downregulation of the AP-1 transcription factors *JUNB, FOS, FOSB,* and *C/EBPbeta* and the genes *LCN2, G0S2, S100A12,* and *S100P* with progression from CP to BC [44].

### 4.2. Chromosomal Abnormalities

Numeric chromosomal changes are 50-fold higher and structural changes are 12-fold higher in patients with BC-CML compared to those with CP-CML [3]. ACAs are present in only 5–10% of patients with CP-CML at diagnosis compared to 50–80% of patients with BC-CML. ACAs are quite heterogenous and they do not have equal pathogenic significance [47]. ACAs are divided into two based on the frequency of the pathways of clonal evolution that are the more common ones: “major route” (+8, i(17q), +19, and +Ph), each occurring in >10% of cases with ACAs, and “minor route” (such as +21, t(3;12), t(4;6), t(2;16), t(1;21), (−Y)). +8 signifies a relatively better prognosis, while 3q26.2 rearrangement or −7/7q− mean a poor response to TKIs and worse survival. However, in the era of TKIs, we do not know how each ACA promotes BC [48].

In the German CML Study IV, 79 of 1151 (6.9%) Ph+ CML patients had ACAs. A total of 38 patients (3.3%) had −Y and 41 (3.6%) had ACAs except −Y. In the latter group, 16 (1.4%) were major-route ACAs and 25 (2.2%) were minor-route ACAs. When patients with standard t(9:22) were compared to patients with major-route ACAs, the 5-year progression free survival (PFS) was 90% vs. 50% and the 5-year OS was 92% vs. 53%, respectively. They concluded that major-route ACAs at diagnosis were associated with a negative impact on survival and signaled progression to AP and BC [20].

Gong et al. evaluated the significance of ACAs in predicting the transformation to BC in 2326 patients treated with TKIs. They stratified the patients into four risk groups (standard, intermediate-1, intermediate-2, and high) and defined patients who were at high risk of progression to BC. They calculated interval 1 (from initial diagnosis to ACA emergence), interval 2 (from ACA emergence to onset of BP), and interval 3 (survival after onset of BP). The median duration of interval 2 was unreached in intermediate-1, 19.2 months in intermediate-2 and 1.9 months in high-risk groups. Interval 2 was the predominant determinant of the risk of BP. They concluded that TKIs were able to mitigate the risk of BC associated with no ACAs or low-risk ACAs but had no effect on the natural course of CML with high-risk ACAs. In addition, they observed a certain risk of myeloid blastic transformation with all of the ACAs (3q26.2, i(17q), −7/7q−, +Ph, and +8) [48].

Wang et al. developed a classification system to reflect the prognostic impact of ACAs in 2013 patients with CML. They stratified the six most common ACAs into two groups. Group 1 included +8, −Y, and an extra copy of the Ph chromosome and had a relatively good prognosis; and group 2 included i(17q), −7/7q−, and 3q26.2 rearrangements and had a relatively poor prognosis. Patients in group 1 had a better treatment response and survival when compared to patients in group 2. When group 1 patients were compared to patients with no ACAs, they had no significant difference in survival [49].

### 4.3. Telomere Biology

Telomeric sequences are synthesized by the enzyme telomerase and telomeres protect chromosomes from end-to-end fusion, degradation, and rearrangement. A telomeric repeat analysis was performed in DNA obtained from the peripheral blood leukocytes of 41 patients with CP-CML and 34 age-matched healthy controls. The average telomere length was expressed as the peak telomere repeat array (TRA). It was reported that the mean peak TRA was 8.7 kb in the healthy controls compared to 6.4 kb in CP-CML patients. When serial samples from 12 CP-CML patients who later progressed to AP and/or BC were analyzed, the mean peak TRA in AP or BC was seen to be 4.1 kb. This reduction in the telomere length was thought to be associated with disease progression in CML [50].

Drummond et al. showed that the degree of telomere shortening correlated with the Hasford prognostic score at diagnosis and proceeded rapidly with disease progression. Patients with AP- or BC-CML had significantly shorter telomeres than patients with CP-CML. In addition, patients with high-risk scores had a significantly greater mean telomere shortening than patients with low-risk scores at initial diagnosis. The rate of telomere shortening during disease progression was 10–20 times the rate in normal neutrophils. It was suggested that telomeric DNA damage in CML could occur by ROS, ineffective repair, and potential sequence loss [51].

There is upregulation of telomerase activity with disease progression in CML. In order to investigate the effect of telomerase inhibition by dominant-negative human telomerase reverse transcriptase (DN-hTERT), DN-hTERT was introduced into BCR-ABL-transformed bone marrow cells and wild-type (WT)-hTERT into their control counterparts. There was a complete inhibition of telomerase activity and a reduction in telomere length in the experimental cells, and they showed signs of apoptosis. These cells also showed enhanced sensitivity to imatinib, which suggests that imatinib and telomerase inhibition might be utilized concomitantly for the treatment of advanced phases of CML [22,52].

### 4.4. Epigenetic Modification

The most commonly occurring mutations in CML seem to be modulators of polycomb repressive complex (PRC) [53]. Polycomb group (PcG) proteins are epigenetic modifiers that assemble in two complexes, PRC2 and PRC1, to modify histones through H3K27 trimethylation and H2AK119 monoubiquitination. They generally repress gene expression [54]. Ko et al. proposed that MBC and LBC transcriptomes were highly congruent, and both underwent PRC-driven epigenetic reprogramming, which was attributed to gain- and loss-of-function mutations in members of the PRC1 and PRC2 complexes, respectively. BC progenitors were enriched for PRC1-related genes and depleted for PRC2-related genes. BMI1 (a core component of PRC1)/PRC1 activity helped to maintain the BC transcriptome, and EZH2 (enzymatic component of PRC2)/PRC2 binding led to DNA hypermethylation-dependent gene repression. By means of epigenetic switching, PRC2 resulted in BC DNA hypermethylation, which silenced key genes involved in myeloid differentiation and tumor suppressor function. On the other hand, PRC1 repressed certain genes, including novel BC suppressors [53,55].

DNA methylation regulates aberrant gene expression in CML. Genes involved in cell cycle regulation (*P16*, *P53*, *PLCD1*, *PER3*, *HIC1*), differentiation (*HOXA4*, *DLX4*, *DDIT3*, *SPI1*), proliferation (*CDH13*, *DAPK1*), apoptosis (*BIM*), Wnt regulation (*sFRP1*, *CBY1*), LSC maintenance (*MTSS1*), and cell signaling (*Jun B*, *SOCS2*) were identified as targets of DNA methylation [56,57]. The number of differentially methylated regions in CP is ~600 CpG sites, while it is ~6500 CpG sites in BC. Heller et al. found that DNA hypermethylation was increased during the progression of CML [58]. In a mouse model of CML, mice transplanted with BCR-ABL leukemic bone marrow had a longer survival with azacitidine than with imatinib or when compared to the untreated mice [59]. In another study, decitabine was used in imatinib-refractory or -intolerant CML. It induced remission via the hypomethylation-mediated clearing of neoplastic cells [60].

Histone modifications are important for gene regulation, and they include acetylation, methylation, and phosphorylation. When there are loss-of-function mutations of CML-related genes, this may cause changes in histone modifications [61]. It was reported that H3K4 demethylase RBP2 expression correlated negatively with BCR-ABL expression. RBP2 could directly downregulate PTEN expression, depending on histone demethylase activity. PTEN targeted the protein phosphatase activity of BCR-ABL, leading to its dephosphorylation. When there is an underexpression of RBP2, it triggers BC by activating the RBP2/PTEN/BCR-ABL cascade [62].

miRNAs are short sequences of conserved non-coding RNAs. They have roles in post-transcriptional gene silencing and regulate some biological activities by binding to their mRNA transcripts, blocking translation, and protein expression. The dysregulation of certain miRNAs has been reported in CML [61].

## 5. Current Treatment Approaches for Myeloid Blast-Phase CML

Despite the advances made in the treatment of CP-CML, the outcome for BC-CML is still poor. Before making a treatment decision, several factors should be taken into consideration [6,63]. The immunophenotype of the blasts is important because the prognosis in MBP-CML is poorer than in LBP-CML, and the former is approximately twice as common than the latter. Immunophenotype is also critical when making treatment decisions about combination therapies because patients with MBP-CML are generally treated with acute myeloid leukemia (AML)-like backbone regimens [63]. The treating physician should also consider if BP is de novo or transformed while the patient was using a TKI. It is recognized that de novo BP has better outcomes [19]. Other factors to consider before making treatment decisions should be the patient’s age, comorbidities, performance status, prior treatment with TKIs, and if there are any BCR-ABL1 kinase mutations [6,10]. The aim should be to bring the patient to CP and then proceed with allogeneic hematopoietic stem cell transplantation (alloHSCT) if the patient is a candidate for transplantation and has a suitable donor [10].

### 5.1. TKI Monotherapy

TKI monotherapy was evaluated in several studies. Nevertheless, the situation is different than in CP-CML, where BCR/ABL is the sole driver mutation and most patients with BC have ACAs. Therefore, response rates to single-agent TKIs in MBC are unsatisfactory [6].

Druker et al. used imatinib at doses ranging from 300 mg to 1000 mg in 58 patients with BC-CML, 38 of which had MBC. A complete hematologic response (CHR) was achieved in four (10.5%) patients and complete cytogenetic response (CCyR) in three (7.9%) patients [64]. In another study with 229 patients with MBC-CML, imatinib was administered at doses of 400 or 600 mg. A sustained CHR was obtained in 18 (7.9%) patients and CCyR in 17 (7.4%) patients [65]. In a phase II trial of imatinib 600 mg daily, 72 of the 92 BC patients had MBC. There was a CHR in 17 (23.6%) patients and CCyR in 5 (6.9%) patients [66].

Cortes et al. conducted a phase 2 trial of dasatinib in patients with imatinib-resistant or -intolerant BP-CML, and 74 of the 116 BC patients had MBC. Dasatinib was administered at a starting dose of 70 mg twice daily and increased to 100 mg twice daily if needed. CHR and CCyR were obtained, respectively, in 18 (24%) and 20 (27%) patients [67]. The two-year follow-up of a randomized, phase 3 study that evaluated the efficacy and tolerability of dasatinib 140 mg once daily and dasatinib 70 mg twice daily was published. The study included 149 patients with MBC. A CHR was achieved in similar percentages of patients treated with 140 mg once daily and 70 mg twice daily of dasatinib (17.3% vs. 17.6%). The CCyR rate in the former group was 14% compared to 21% in the latter group. The results supported the use of dasatinib 140 mg once daily for the BP-CML indication [68].

Nilotinib was also used in the treatment of patients with BP-CML in published studies. We note that it is not FDA-approved for use in BP-CML. In one study of 136 patients with BP, 105 had MBC and they were treated with nilotinib 400 mg twice daily. CHR and CCyR were obtained in 24% and 30% of patients, respectively, and the median OS was 10.1 months [69]. The ENACT study was an expanded access study and included 190 patients with BC-CML, 133 of whom had MBC. Nilotinib was administered as 400 mg twice daily. There was CHR in 9 (6.8%) patients and CCyR in 11 (8.3%) patients [70].

A phase 1/2 study of bosutinib included 23 MBC patients in a total of 64 patients with BC. The starting dose of bosutinib was 500 mg once daily and dose escalation to bosutinib 600 mg once daily was permitted for lack of efficacy if there was no bosutinib-related grade 3–4 toxicity. Bosutinib was used as second-line therapy in 36 patients with BC and as third- or higher-line therapy in 28 patients. Although they did not specify the response rates in patients with MBC, the response rate in the overall population of patients with BC-CML was reported. The CHR and CCyR rates in the group with one prior line of therapy were 27% and 37% compared to 4% and 17% in the group with two or more prior lines of therapies, respectively [71].

The PACE trial of treatment with ponatinib included 62 patients with BC and 52 of them had MBC. Ponatinib was administered at a starting dose of 45 mg once daily. There was a 29% rate of major hematologic response (MaHR) and 15% CCyR in patients with MBC [72].

Briefly, it can be concluded that single-agent TKIs for the treatment of MBC result in low response rates. The median OS was less than 12 months in all studies, and only a very small percentage of patients could proceed with alloHSCT [6]. Other BCR-ABL1 TKIs, such as asciminib and olverembatinib, are being investigated at different stages of CML; however, efficacy data supporting their use are not available at this time [63].

### 5.2. Combination Therapy

As the results of treatment with TKI monotherapy in MBC are inadequate, younger patients without significant comorbidities should be considered for AML-based intensive chemotherapy regimens combined with a TKI. Older patients or those with comorbidities may be eligible for lower intensity regimens, like a hypomethylating agent (HMA) plus venetoclax, combined with a TKI [11]. A summary of various chemotherapy regimens administered with or without TKIs for patients with MBP-CML is provided in Table 2.

In a phase I/II trial of 16 patients with MBC, imatinib 600 mg/day was administered with mitoxantrone, etoposide, and cytarabine (MEC). In two of the four cohorts, imatinib was administered starting from day 15, and in the other two cohorts, it was started from day 1. A hematologic response was obtained in 13 (81.2%) patients. Six patients (37.5%) underwent alloHSCT. The median OS was 6.4 months [73].

In another study, imatinib 600 mg/day was combined with low-dose cytarabine (10 mg/day) and idarubicin in 19 patients with MBC. A CHR was achieved in nine (47.3%) and CCyR in three (15.8%) patients, and six (31.6%) patients received alloHSCT. The median survival was 5.8 months [74].

Deau et al. conducted a dose escalation study of daunorubicin combined with imatinib 600 mg/day and cytarabine 200 mg/day, followed by HSCT or maintenance therapy with imatinib in 36 patients with MBC. A CHR was achieved in 20 (55.5%) and CCyR in 11 (30.6%) patients. Eleven (30.6%) patients underwent alloHSCT. The median OS was 16 months. The combination of imatinib with the 3 + 7 regimen yielded high response rates [75].

One small series of four patients who developed BC on imatinib included two patients with MBC, one with biphenotypic leukemia, and one with lymphoid leukemia. All patients were treated with FLAG-IDA combined with dasatinib 100 mg/day. All patients had morphological remission, three had CCyR, three underwent alloHSCT, and one was scheduled to undergo alloHSCT [76].

MATCHPOINT was a single-arm, multicenter, phase 1/2 trial conducted in the UK. It included 17 patients with BC; 9 were diagnosed with MBC, 4 with LBC, and 4 with mixed leukemia. Patients were administered one or two cycles of FLAG-IDA with ponatinib 30 mg/day and eligible patients underwent alloHSCT. Among patients with MBC, one (11.1%) achieved CHR, four (44.4%) CCyR, and six (66.7%) proceeded to alloHSCT. The median OS for all patients with BC was 12 months. Also, post-transplant, ponatinib at 15 mg/day was administered to patients with major molecular response (MMR) and the safety profile appeared to be as previously reported [77].

As epigenetic changes are known to play a role in the pathogenesis of MBC, some studies combining hypomethylating agents with a TKI have been published [10]. Oki et al. included 28 patients (10 with MBC) into their study of decitabine and imatinib 600 mg/day. CHR and CCyR were each achieved in only two (20%) patients. The median OS was 3.5 months [78].

In another trial, five patients with MBC were treated with azacitidine combined with dasatinib or nilotinib. Four patients were ineligible for and the fifth was refractory to intensive chemotherapy. All of them obtained CHR and two of them had CCyR. Two of the patients underwent alloHSCT. With a follow-up of 24 months, four patients were reported to be alive [79].

In another trial of 16 advanced-phase CML patients treated with azacitidine combined with dasatinib, nilotinib, or ponatinib, there were seven patients with MBC. The reported CCyR was 42.9% and the median OS was 27.4 months [80].

In a prospective study of decitabine with dasatinib 100 mg/day, 19 patients with BC, 18 of whom had MBC were treated. There were 17 patients who were evaluable for response, and 7 (41.2%) achieved a CHR and CCyR. Overall, eight patients (26.7%) in this study were bridged to alloHSCT. The authors commented that the decitabine with dasatinib combination was a safe alternative in this population and that the efficacy results were better than those expected with each agent alone [81].

Saxena et al. analyzed the response rates and survival outcomes of 104 patients with MBC who were treated with the four following frontline treatment modalities: intensive chemotherapy (IC) + TKI (20), hypomethylating agent (HMA) + TKI (20), TKI alone (56), or IC alone (8). We summarize their results from the IC + TKI and HMA + TKI arms. In the IC + TKI arm, dasatinib was used in 10 patients (50%), ponatinib in 7 (35%), nilotinib in 2 (10%), and bosutinib in 1 patient (5%). In this arm, a CCyR was achieved in eight (40%) patients and the median OS was 12.9 months. Seven patients (35%) proceeded to alloHSCT. In the HMA + TKI arm, dasatinib was used in 11 patients (55%), imatinib in 7 (35%), and nilotinib and ponatinib in 1 patient each (5%). In this arm, a CCyR was achieved in 10 (50%) patients and the median OS was 10.1 months. Six patients (30%) underwent alloHSCT. The five-year OS rates were 30% in the IC + TKI arm and 28% in the HMA + TKI arm [12].

A retrospective analysis of 16 patients (9 MBC and 7 Ph+ ALL) treated with venetoclax + TKI combined with decitabine or IC was performed. Five of the patients with MBC (56%) were treated with decitabine-based regimens and four (44%) were treated with IC-based regimens. The TKI was ponatinib in four patients (44%), dasatinib in three (33%), and nilotinib or bosutinib in one patient each (11%). A CCyR was obtained in three patients (33.3%), and two patients (22.2%) had HSCT. Venetoclax + TKI-based regimens showed encouraging activity in heavily pretreated patients with MBC [82].

With the limitations of cross-trial comparisons and a lack of randomized controlled trials, the combination of TKIs with intensive chemotherapy or lower-intensity chemotherapy appeared to achieve better results than with TKIs alone. Still, more effective treatment options are needed in patients with MBC. Based on the available data, NCCN guidelines recommend clinical trials or AML-type induction chemotherapy combined with a TKI for the initial treatment of MBC. For eligible patients in remission, the recommendation is alloHSCT. For non-candidates for allo-HSCT, consolidation chemotherapy and TKI maintenance is recommended [17].

### 5.3. Other Therapies

Asciminib is the most recently approved TKI, and it binds to the myristoyl pocket of BCR-ABL1 in contrast to other TKIs, which bind to the ATP-binding site. In a phase I dose-finding study, asciminib was combined with imatinib, dasatinib, or nilotinib in 326 patients with relapsed, refractory, or intolerant CP-, AP-, or BP-CML, or Ph+ ALL (NCT02081378 [85]). Results from this study are pending.

We searched clinicaltrials.gov for trials in patients with MBP-CML that are currently recruiting or active but not recruiting (Table 3). Four of the trials specifically included MBP-CML in their inclusion criteria and two others listed BP-CML.

One of the recruiting trials is the PONAZA trial, which is a phase 2 trial combining azacitidine with ponatinib 45 mg/day. If a CHR is obtained during induction, which is the first three cycles, ponatinib will be decreased to 30 mg/day. If MMR is reached, ponatinib will be decreased to 15 mg/day (NCT03895671).

Olverembatinib (HQP1351) is an oral, third-generation BCR-ABL1 TKI that is under investigation for the treatment of CML and other indications. Olverembatinib is not FDA-approved, but is currently approved in China for the treatment of adult patients with TKI-resistant CP-CML or AP-CML harboring the T315I mutation, as confirmed by a validated diagnostic test. It binds to the ATP-binding site and has demonstrated activity against the T315I mutation and some other mutants [92]. In a recruiting trial, olverembatinib 40 mg every other day is being administered with decitabine to patients with newly diagnosed or R/R myeloid AP or MBP-CML (NCT05376852).

In one trial, investigators are adding venetoclax to cladribine, idarubicin, and cytarabine for patients with various myeloid malignancies, including those with newly diagnosed or R/R MBP-CML (NCT02115295). The planned enrollment is reported to be 508 patients, and they report results from 50 patients, though no patients with MBP-CML have been enrolled [93].

In one phase 2 trial, decitabine and venetoclax are being combined with ponatinib 45 mg/day in MBP-CML, AP-CML, and Ph+ AML patients. Ponatinib is reduced to 30 mg for patients in CR/CRi and to 15 mg/day for patients in complete molecular response (NCT04188405). They reported results for 15 patients, of whom 10 had MBP-CML, 4 AP-CML, and 1 Ph+ AML. When all 15 patients were considered, CR/CRi was obtained in six (40%), morphologic leukemia free state (MLFS) in five (33%), and four patients underwent HSCT. At a median follow-up of 9.8 months, the median OS was 11 months [94].

Hu8F4 is an investigational humanized T-cell receptor-like monoclonal antibody that binds to the conformational epitope of PR1 bound to HLA-A2. It was used in a first-in-human dose escalation trial in patients with R/R AML, MDS, chronic myelomonocytic leukemia (CMML), and MBP-CML (NCT02530034). Results from ten patients with R/R AML were reported, but no patients with MBP-CML were enrolled [95].

Vodobatinib is an investigational third-generation TKI and it is being evaluated in a phase 1/2 study in patients with resistance or intolerance to CML (NCT02629692). In an abstract published in 2021, they reported on 52 patients enrolled in dose expansion. There were four patients with BP-CML; two achieved CHR and two had disease progression [96].

The number of trials recruiting patients with MBC is relatively low and the number of patients in each trial is also low. To evaluate the efficacy and safety of new treatment regimens, well-planned randomized trials with a sufficient number of patients should be considered, particularly for combination approaches.

### 5.4. Allogeneic Hematopoietic Stem Cell Transplantation

With the currently available treatments for CP-CML, alloHSCT is rarely needed in patients with CP-CML. However, patients with BC have multiple mutations, and up to 80% have ACAs. Although TKIs have improved survival to some extent, the one curative option in patients with BP-CML remains alloHSCT [63].

In a retrospective European Society for Blood and Marrow Transplantation (EBMT) study, 170 patients who underwent alloHSCT for BP-CML after TKI pretreatment were analyzed. Eighty-eight (52%) patients had MBC. For patients who were in remission at the time of transplant, risk factors for inferior survival included advanced age (≥45 years), a lower performance status (≤80%), longer interval from diagnosis of BC to transplant (>12 months), myeloablative conditioning, and unrelated donor transplant. A multivariable analysis of the entire cohort showed that active BC at the time of transplant was the strongest factor associated with a shorter OS (HR, 1.87; *p* = 0.10). For patients with active BC at the time transplant, only unrelated donor transplant was significantly associated with longer leukemia-free survival and there was a trend to improved OS [97].

A Center for International Blood and Marrow Transplant Research analysis of 449 alloHSCTs performed between 1999 to 2004 in patients with advanced phase CML revealed that disease-free survival was better for patients when they were transplanted in the second chronic phase compared to those transplanted in AP or BP (35–40% vs. 26–27% vs. 8–11%). At the time of the conditioning regimen, 184 patients were in CP, 185 patients were in AP, and 80 patients were in BP (42 were in MBC) [98].

In another study, long-term outcomes and risk factors for 96 patients with BC and 51 patients with AP transplanted between 1990 and 2018 were reported. The 15-year OS was 34% and PFS was 26%. The cumulative incidences of non-relapse mortality and relapse were 28% and 43%, respectively. The results of the study showed that being in a non-BC phase before HSCT was an advantage [99].

Nicolini et al. reported on 63 patients with MBC or LBC who underwent a first alloHSCT. The median OS was 22 months. The transplant-related mortality was significantly negatively influenced by the EBMT score and BC status at diagnosis. OS and PFS were improved by disease control prior to transplant (second CP was better than persistent BC) [100].

In a retrospective analysis of 104 patients with MBC, patients who underwent HSCT (n = 19) had a superior OS compared to those that did not (n = 22), and the 5-year OS rates were 58% vs. 22% (*p* = 0.12), respectively [12].

In conclusion, the long-term outcomes of BC-CML with alloHSCT are not satisfactory; however, they are still superior to non-transplant options. Patients seem to fare better when they are transplanted in the second chronic phase. The use of maintenance TKIs after alloHSCT is inconclusive [11]; however, it is frequently used in clinical practice if patients have no graft-versus-host disease or significant cytopenias. There is unmet medical need for more effective treatments in BC-CML.

## 6. Possible Mechanisms of Resistance to Tyrosine Kinase Inhibitors

Although TKIs successfully induce hematologic, cytogenetic, and molecular responses in patients with CP-CML, LSCs in CML are independent of the activity of BCR-ABL1 and they are responsible for progression to BP-CML [101].

TKI resistance can occur in one of two ways. The first is BCR-ABL1-dependent resistance, which includes mechanisms that prevent the effective inhibition of BCR-ABL1, leading to decreased TKI activity. Using an alternative TKI would be the way to combat BCR-ABL1-dependent resistance. The second is BCR-ABL1-independent resistance that occurs through alternative survival pathways in spite of an effective BCR-ABL1 inhibition. Changing the TKI would not be expected to work in this scenario. Although both mechanisms contribute to overt clinical resistance, acquired resistance is usually BCR-ABL1-dependent and primary resistance is usually BCR-ABL1-independent [102,103].

In this section, we discuss the various mechanisms by which BCR-ABL1-dependent and BCR-ABL1-independent resistance lead to ineffective treatment with TKIs.

### 6.1. BCR-ABL1-Dependent Resistance

#### 6.1.1. BCR-ABL1 Kinase Domain Mutations

Patients who do not have an optimal response to imatinib have been observed to have mutations in the BCR-ABL1 kinase domain. These mutations are the cause of resistance to TKIs in about two-thirds of patients with AP- and BC-CML [104,105]. To date, >100 different mutations have been defined [104]. In the IRIS study that compared interferon-alpha + cytarabine with imatinib monotherapy, most patients with advanced-phase CML rapidly became resistant to imatinib [106].

The kinase-domain mutations change the conformation of the ABL kinase domain and hinder the binding of TKIs [104]. Mutations that cause resistance to imatinib can affect one of four sites, which are the ATP-binding loop (P-loop) (E255K, Q252H, Y253F), the imatinib-binding site (F317I/L, T315I), the catalytic domain (C-loop) (M351T), and the activation loop (A-loop) (H396P) [107,108]. Mutations in the first three sites are more common [105].

The T315I mutation is the most common mutation, and it is found in 4–15% of patients with CML who are resistant to imatinib. It is at the imatinib-binding site and leads to the replacement of threonine with isoleucine at position 315. The treatment of patients with this mutation poses a problem because patients are resistant to second-generation TKIs [104]. E255K and M351T comprise 9–14% of mutations. Y253F, Q252H, F317L, and other mutations comprise 3–6% of mutations [107].

Compound mutations and polyclonal mutations pose another challenge. Compound mutations occur when there are ≥2 point mutations within the same BCR-ABL1 allele and polyclonal mutations arise when there are ≥2 point mutations in the kinase domain of separate BCR-ABL proteins [104,107]. The sequential use of TKIs may be one of the reasons for the increase in compound mutations [104]. Although a single T315I mutation responds to treatment with ponatinib, most compound mutations containing T315I are resistant to ponatinib [109].

#### 6.1.2. Mutations Outside the BCR-ABL1 Kinase Domain

Not commonly, there may be mutations in the SH2, SH3, and Cap domains that lead to the autoinhibition of ABL kinase. These mutations together with other mutations might destabilize the inactive BCR-ABL conformation [104].

Asciminib is an allosteric inhibitor that inhibits the myristoyl-binding site and is effective in kinase domain mutations. However, mutations against sites that compose the hydrophobic allosteric pocket have been defined and they can cause resistance to allosteric inhibitors [105].

#### 6.1.3. BCR-ABL1 Gene Overexpression

The BCR-ABL1 gene may be overexpressed because of genetic, transcriptional, or post-translational mechanisms. It was observed that an increased BCR-ABL1 expression in cell lines was associated with resistance to TKIs [110].

In one study, the granulocyte–macrophage progenitor pool from patients with BC-CML and imatinib-resistant CML was expanded. It was observed that they expressed BCR-ABL, and the levels of nuclear β-catenin were higher than progenitors in the bone marrow. The activation of the β-catenin pathway in CML granulocyte–macrophage progenitors was interpreted as an enhancement in self-renewal activity and the leukemic potential of the cells [111].

In another study, it was seen that cells that expressed high levels of BCR-ABL, like in BC, were much less sensitive to imatinib. It took a shorter time for them to yield a mutant subclone resistant to TKIs than cells with low expression levels of BCR-ABL, as in CP-CML [26]. Therefore, when BCR-ABL1 levels are high, kinase activity will persist in spite of TKI usage, leading to the survival of leukemia cells. The leukemia cells will then acquire kinase domain mutations, and this will cause even more resistance [102].

#### 6.1.4. DNA Damage Repair and Gene Instability

ROS can accumulate because of abnormally active tyrosine kinase activity. ROS levels are significantly higher in BP-CML cells than in CP-CML cells. Their accumulation may cause DNA damage, double-stranded breaks in DNA, and mismatch repair. Genomic instability and subsequent mutations, chromosomal translocations, and deletions induced by ROS may cause drug resistance [107].

### 6.2. BCR-ABL1-Independent Resistance

Although BCR-ABL1-dependent resistance mechanisms are the most common cause of resistance to TKIs, BCR-ABL1-independent mechanisms of resistance occur in about 40% of relapsed patients with CML [112]. Under these circumstances, CML cells can survive through leukemia cell-intrinsic mechanisms or leukemia cell-extrinsic factors, which are mediated by the bone marrow microenvironment [113].

#### 6.2.1. Alternative Pathways

One major BCR-ABL1 downstream signaling pathway that plays a role in proliferation, survival, and drug resistance in CML is the JAK/STAT signaling pathway, and its important effectors are STAT3 and STAT5 [114]. When STAT3 levels are reduced, the sensitivity of K562 human CML cells to imatinib-induced cell death is enhanced [102,115].

After the activation of PI3K, AKT is phosphorylated, which leads to the avoidance of apoptosis and cell cycle progression [107]. Burchert et al. demonstrated that the treatment with imatinib of BCR/ABL-positive LAMA-84 cells and primary leukemia cells in vitro, and in a patient with CP-CML in vivo, it activated the phosphatidylinositol 3-kinase (PI3K)/AKT/mammalian target of the rapamycin (mTOR) pathway [116].

The mitogen-activated protein kinase (MAPK) pathway (RAF/RAS/mitogen-activated protein kinase (MEK)/extracellular signal-regulated kinase (ERK) pathway or RAS pathway) is a major downstream signaling pathway in BCR-ABL1+ cells. When there is the activation of the RAS pathway by mechanisms independent of BCR-ABL1 signaling, this can lead to resistance to TKIs in CML [114].

One recent study showed that the noncanonical NF-κB pathway was activated by the previously uncharacterized protein, FAM167A. The authors reported that the FAM167A/desmoglein-1 (DSG1) axis regulated the noncanonical NF-κB pathway and controlled BCR-ABL-independent resistance in CML cells derived from CML patients [117].

Osimertinib, a third-generation covalent EGFR inhibitor, was shown to induce the apoptosis of the CD34^+^ leukemia stem and progenitor cells in EGFR-negative AML and CML, but no comparable cell death occurred in CD34^−^ cells. The authors stated that CML LSCs were independent of BCR-ABL for survival, and osimertinib could selectively kill CD34^+^ cells. They concluded that osimertinib could act synergistically with imatinib to target the BCR-ABL-independent mechanism of resistance [118].

#### 6.2.2. Epigenetic Dysregulations

It has been reported that progression to BC is characterized by the emergence of TKI-resistant clones with perturbations in PRC-related pathways. It can be said that prolonged, unopposed BCR-ABL1 activity enhances PRC2 activity in CP-CML, which leads to the methylation of PRC2-bound genes [55]. EZH2 is a histone methyltransferase that provides the catalytic subunit of PRC2, and it was shown to be overexpressed in CML leukemia-initiating cells (LICs) [102,119]. When EZH2 is inactivated in mice and through CRISPR/Cas9-mediated gene editing, the disease initiation, maintenance, and survival of LICs is prevented and extended survival is observed, irrespective of BCR-ABL1 mutational status [119].

Patients with drug-resistant and -intolerant CML have a higher number of methylated genes. Histone modifications leading to gene transcription and silencing can also contribute to drug resistance [119].

#### 6.2.3. Changes in Drug Influx/Efflux Pumps

Drug resistance may occur because of changes in the expression level and function of drug transporters [107]. The key transporter for imatinib uptake is the membrane influx pump, human organic cation transporter 1 (hOCT1). When the OCT1 content is high, this is predictive of MMR; however, low OCT1 is associated with a suboptimal response [104,107]. The ATP-binding cassette subfamily member 1 (ABCB1) (P-glycoprotein or MDR1) and ATP-binding cassette subfamily member 2 (ABCB2) (BCRP) are the efflux pumps. When there is ABCB1 overexpression, long-term outcomes can be poor and progression to BC is more likely. BCRP is found in LSCs and protects them from the action of TKIs [107].

#### 6.2.4. Bone Marrow Microenvironment

The bone marrow is composed of hematopoietic and mesenchymal-derived cells. The bone marrow stroma is made up of mesenchyme-derived cells, extracellular matrix, and macrophages. One contributor to drug resistance in CML may be the dysregulated interactions between hematopoietic cells and stromal cells. The interactions between these two types of cells are bidirectional and one may be able to influence the other [107].

The CXCL12-CXCR4 axis is important in sustaining LSCs. There is the overexpression of the CXCL12 receptor CXCR4 in CML, and this provides an increased proliferative capacity and nilotinib resistance in vitro [120]. BCR-ABL activity leads to altered CXCR4 expression, which causes a defective adhesion of CML cells to bone marrow stroma. With the imatinib treatment, CXCR4 and BCL-XL expression is increased, and cells that migrate to the bone marrow are protected from drug-induced cell death [104]. In one study using a mouse model of progressive disease, the CXCR4 antagonist plerixafor combined with nilotinib reduced leukemia burden significantly below the baseline level suppression by nilotinib alone [121].

Integrins take part in cell adhesion-mediated drug resistance. The β_1_ integrins VLA4 and VLA5 on CML cells can bind to VCAM-1 and fibronectin on bone marrow stromal cells and extracellular matrix, and they act upon several genes, which leads to drug resistance [104].

The Wnt-β-catenin pathway is also important in the pathogenesis of CML [113]. It has been reported that granulocyte–macrophage progenitor cells from patients with MBP-CML could acquire self-renewal capacity through nuclear β-catenin signaling. Despite BCR-ABL1 inhibition by imatinib, leukemia cells maintained their cytoplasmic β-catenin expression, which may contribute to intrinsic TKI resistance in CML cells [111]. Increased cytoplasmic N-cadherin-β-catenin complex formation, together with enhanced β-catenin nuclear translocation and transcriptional activity, causes the N-cadherin-mediated adhesion of CML progenitors to mesenchymal stem cells. The interaction between N-cadherin and the Wnt-β-catenin pathway protects CML cells from the effect of TKIs [122].

## 7. Conclusions

TKIs have drastically improved outcomes for patients with CP-CML. However, treatment outcomes in BP-CML have not greatly improved. MBC is a rare, rapidly progressive disease, and it is characterized by genetic and chromosomal abnormalities, telomere shortening, and epigenetic modifications. Patients with MBC have worse treatment outcomes than patients with LBC, and treatment response to TKI monotherapy is poor. The combination of TKIs with intensive chemotherapy or lower intensity therapies appears to yield better treatment outcomes than TKIs alone. Although TKIs are critical for the treatment of MBC, both BCR-ABL1-dependent and -independent mechanisms play roles in the development of resistance to TKIs. As these mechanisms are heterogenous, it is hard to achieve success by utilizing only one treatment approach. Therefore, the treatment of MBC should be targeted towards both BCR-ABL1-dependent and -independent mechanisms. However, challenging, randomized clinical trials should ultimately be conducted to adequately evaluate novel treatments for patients with MBC, with the hope to improve future outcomes.

## Figures and Tables

**Table 1:** Select biologic events that play roles in progression to BP-CML.

	Abnormality	Mechanism
**Genetic events**		
	BCR-ABL1 kinase domain, RUNX1, GATA, UBE2A	Mutation
	p53	Genetic or functional inactivation
	EVI1, c-MYC, SOCS2, CD52, MHC class II complex-related genes, CD34	Upregulation
	IKZF1	Deletion
	PP2A	Inactivation
	MPO	Downregulation
**Chromosomal abnormalities**		
	Major route (+8, i(17q), +19, and +Ph)	-
	Minor route (+21, t(3;12), t(4;6), t(2;16), t(1;21), (−Y))	-
Telomeres		
	Telomere length	Reduction
	Telomerase activity	Reduction
**Epigenetic modifications**		
	DNA methylation	Upregulation or downregulation
	Histone modification	Upregulation
	miRNA	Dysregulation

**Table 2:** Various chemotherapy regimens with/without TKIs studied in patients with MBC *.

Author	# of Patients	Combination Treatment	HR ** (%)	CHR ** (%)	CCyR (%)	AlloHSCT (%)	Median OS (Months)
Fruehauf et al. [73]	16	Mitoxantrone, Etoposide, Cytarabine + Imatinib	81.2	NA	NA	37.5	6.4
Quintás-Cardama et al. [74]	19	Low-dose Cytarabine, Idarubicin + Imatinib	73.7	47.3	15.8	31.6	5.8
Deau et al. [75]	36	Daunorubicin, Cytarabine (3 + 7) + Imatinib	77.7	55.5	30.6	30.6	16
Milojkovic et al. [76]	4 ^a^	Fludarabine, Cytarabine, Idarubicin, G-CSF (FLAG-IDA) + Dasatinib	100	NA	75	100	NA
Copland et al. [77]	9	Fludarabine, Cytarabine, Idarubicin, G-CSF (FLAG-IDA) + Ponatinib	NA	11.1	44.4	66.7	12 ^b^
Oki et al. [78]	10	Decitabine + Imatinib	30	20	NA	NA	3.5
Ghez et al. [79]	5	Azacitidine + Dasatinib or Nilotinib	NA	100	40	20	NR
Ruggiu et al. [80]	7	Azacitidine + Dasatinib or Nilotinib or Ponatinib	71.4	NA	42.9	NA	27.4
Abaza et al. [81]	18	Decitabine + Dasatinib	76.4	41.2	41.2	26.7 ^c^	13.8 ^c^
Saxena et al. [12]	40	IC + TKI (20) HMA + TKI (20)	60 ^d^ 55 ^d^	NA	NA	35 30	12.9 10.1
Maiti et al. [82]	9	IC or Decitabine + Venetoclax + TKI	NA	NA	33.3	22.2	10.9
Fang et al. [83]	12	G-CSF + HHT + Imatinib	91.7	58.3	25	75	NA
Maiti et al. [84]	3	HHT + Imatinib	NA	66.7	33.3	NA	NA

* All of the regimens listed in the table constitute off-label or investigational use. ** The definitions of HR and CHR are not uniform across trials; however, in general, HR refers to some form of hematological improvement and CHR refers to a normal complete blood count, peripheral blood film, and no splenomegaly on physical exam. HR: Hematologic response; CHR: Complete hematologic response; CCyR: Complete cytogenetic response; AlloHSCT: Allogeneic hematopoietic stem cell transplantation; OS: Overall survival; NA: Not available; G-CSF: Granulocyte colony-stimulating factor; FLAG-IDA: Fludarabine, cytarabine, idarubicin, and G-CSF; IC: Intensive chemotherapy; TKI: Tyrosine kinase inhibitor, HMA: Hypomethylating agent; HHT: Homoharringtonine. ^a^ The results for this study are for all 4 patients in the study (2 MBC, 1 LBC, and 1 biphenotypic leukemia). ^b^ Median OS result is reported for all 17 patients in the study (9 patients with MBC, 4 patients with LBC, and 4 with mixed leukemia). ^c^ Percentage of patients who proceeded to alloHSCT and median OS results are reported for all 30 patients in the study (19 patients with BC, including 18 with MBC and 1 with LBC, 7 patients with AP-CML, and 4 with Ph+ AML). ^d^ The rates reported reflect the rates of CR/CRi.

**Table 3:** Currently recruiting and active, not recruiting clinical trials in adult patients with MBP-CML (trials on HSCT are excluded) listed on clinicaltrials.gov (accessed on 16 September 2024).

NCT Number & Trial Name	Phase	Drugs	Patient Populations	Estimated Enrollment	Primary Endpoint	Status
NCT03895671 [86] Ponaza: A combination of ponatinib and 5-azacitidine in CML in AP or in MBP	2	Azacitidine Ponatinib	*First-line* MBP-CML AP-CML	40	OS (2 yr)	R
NCT05376852 [87] Decitabine and HQP1351-based chemotherapy regimen for the treatment of advanced CML	2	Decitabine Olverembatinib (HQP1351)	*First line, R/R* MBP-CML MAP-CML	40	CR/CRi (12 wk)	R
NCT02115295 [88] Cladribine, idarubicin, cytarabine, and venetoclax in treating patients with AML, HR-MDS, or BP-CML	2	Cladribine Idarubicin Cytarabine Venetoclax	*First line, R/R* MBP-CML AML BPAL HR-MDS	508	CR (by 12 mo)	R
NCT04188405 [89] Decitabine, venetoclax, and ponatinib for the treatment of Ph+ AML or MBP or AP-CML	2	Decitabine Venetoclax Ponatinib	*First line, R/R* MBP-CML AP-CML Ph+ AML	30	CR/CRi (8 wk)	Active, NR
NCT02530034 [90] Hu8F4 in treating patients with advanced hematologic malignancies	1	Hu8F4	*R/R* BP-CML HR-MDS CMML AML HR-MF	72	DLTs, minimum safe and biologically effective dose	Active, NR
NCT02629692 [91] Safety and anti-leukemic activity of vodobatinib (K0706) for the treatment of resistant/intolerant Ph+ CML to ≥3 prior CML therapies	1, 2	Vodobatinib	*R/I* CP-CML AP-CML BP-CML	122	MTD AEs MaHR ^a^ NEL ^a^	Active, NR

ALL: Acute lymphoblastic leukemia; AML: Acute myeloblastic leukemia; BPAL: Biphenotypic acute leukemia; CML: Chronic myeloid leukemia; CMML: Chronic myelomonocytic leukemia; HR: High risk; MDS: Myelodysplastic syndrome; MF: Myelofibrosis; Ph: Philadelphia chromosome; PLL: Prolymphocytic leukemia; CP: Chronic phase; AP: Accelerated phase; BP: Blastic phase; AP: Accelerated phase; MBP: Myeloid blast phase; LBP: Lymphoid blast phase; R/R: Relapsed/refractory; R/R/I: Relapsed/refractory/intolerant; CR: Complete remission; CRi: Complete remission with incomplete count recovery; MaHR: Major hematologic response; NEL: No evidence of leukemia; OS: Overall survival; DLT: Dose-limiting toxicity; MTD: Maximal tolerated dose; R: Recruiting; NR: Not recruiting. ^a^ MaHR and NEL will be determined for subjects with BP-CML at study entry.
